# Supplementary material for: Virus-Induced Gene Silencing (VIGS): A Powerful Tool for Crop Improvement and Its Advancement towards Epigenetics
Source: Int J Mol Sci. 2023 Mar 15;24(6):5608. doi: 10.3390/ijms24065608 (PMC10057534; doi:10.3390/ijms24065608)
Supplement: Supplementary file 1 [file ijms-24-05608-s001.zip › ijms-2177484-supplementary.pdf]

Table S1: VIGS Vectors, Plant Species, and Their Method of Transmission Used in Gene Silencing

Virus Vector	Silenced Host	Group/Genus	Vector Transmission Method	Gene Silenced	Nature of Genome	References
			**RNA Viruses**			
**Tobacco mosaic virus**	*Nicotiana benthamiana, Nicotiana tabacum*	Tobamovirus	Rub inoculation	*pds, psy, rbcS, chsA*	Positive-strand RNA, monopartite	[1]; [2]; [3]
**Apple latent spherical virus (ALSV)**	*Nicotiana* spp., *Malusdomestica*, *Pyrus communis L*, *Solanum lycopersicum, N. occidentalis, n. glutinosa, Cucumis* spp, cucurbit species, several legume species, pumpkin (*Cucurbita maxima*), *Arabidopsis thaliana*	Cheravirus	sap inoculation, and particle bombardment	*pds, su, pcna, sulfur* gene	Positive-strand RNA, bipartite	[4]; [5]; [6]
**Brome mosaic virus**	*Oryza sativa, Hordeum vulgare, N. benthamiana, Sorghum bicolor, Zea mays, Triticum aestivum* L, *Festuca arundinacea*	Bromovirus	Rub inoculation, vacuum infiltration, and Agro infiltration	*Ubiq, ChlH, PDS actin 1, rubisco activase, HSP70-1, PHYTOENE DESATURASE* (*Ta PDS*) and *PHOSPHATE 2* (*TaPHO2*)	Positive-strand RNA, tripartite	[7]; [8]; [9]; [10]; [11]
**Barley stripe mosaic virus**	*Brachypodium distachyum, Hordeum vulgare L*, *Avena* species and *Triticum aestivum*	Hordeivirus	Rub inoculation	*pds, Bx1,* Ta_PYL4AS_A	Positive-strand RNA, tripartite	[12]; [13]; [14]; [15]; [16]; [16]
**Bean pod mottle virus**	*Glycine max,* Kefeng 1 and Phaseolus *vulgari,*	Comovirus	Rub inoculation, Mechanical inoculation, and particle bombardment	*Pds,* GmCAL	Positive-strand RNA, bipartite	[17]; [18]; [19]; [20]

Table S1

**Cucumber mosaic virus**	*Glycine max*, Zea *mays*	Cucumovirus	Sap inoculation, Mechanical inoculation	*chs, sf3'h1,* isopentenyl/dimethylallyl diphosphate synthase *(ZmIspH)*	Positive-strand RNA, tripartite	[21]; [22]
**Cymbidium mosaic virus (CymMV)**	*Phalaenopsis orchids, Phalaenopsis* flower	Potexvirus	Rub inoculation, Agro infiltration	*PeMADS*6, *PeUFGT3*, *PeMADS5* and *PeMADS 6*	*Positive Single-stranded* (+ssRNA) genome	[23]; [24]
**Potato Virus X (PVX)**	*N.benthamiana Solanum tuberosum,*	Potexvirus	Agro inoculation	*gus, pds, DWARF, SSU, NFL, LFY*	Positive-strand RNA, monopartite	[25]; [26]; [27]
**Potato Virus A (PVA)**	*N.benthamiana*	Potyvirus	Rub inoculation and particle bombardment	Systemic *gfp* transgene silencing	*Single-stranded,* RNA viruses	[28]
**Watermelon mosaic virus (WMV)**	*Cucumis melo*	Potyvirus	Agrobacterium-mediated gene transfer	Melon Phytoene desaturase (*PDS*)	*Single-stranded,* RNA viruses	[29]
**Zucchini yellow mosaic virus (ZYMV)**	*Cucurbita pepo*	Potyvirus	Agrobacterium-mediated gene transfer	Bacterial phytoene synthase (*crtB*)	*Single-stranded*, RNA viruses	[29]
**Pea early browning virus**	*Lathyrus odorata, Medicago truncatula*	Tobravirus	Agro inoculation	*pds, uni, kor*	Positive-strand RNA, bipartite	[30]
**Poplar mosaic virus (PopMV)**	*N.benthamiana*	Carlavirus	Agro inoculation	*gfp*	Positive-strand RNA, monopartite	[31]
**Plum pox virus (PPV)**	*N.benthamiana*	Potyvirus	Agro inoculation	IBDV VP3 inhibits PKR-dependent apoptosis	+ssRNA	[32]

**Sunn-hemp mosaic virus (SHMV)**	*N.benthamiana, Medicago truncatula*	Tobamovirus	Rub inoculation	*gfp, PDS, CH42*	Positive-strand RNA virus	[33]
**Tobacco necrosis virus A (TNV-A)**	*N.benthamiana*	Necrovirus	Agro inoculation	Non-putative gene	+ssRNA	[34]
**Tomato bushy stunt virus (TBSV)**	*N.benthamiana*	Tombusvirus	Rub inoculation	*RMJ3, T33*	+ssRNA	[35]
**Tobacco rattle virus (TRV)**	Opium or California poppy, *N. benthamiana, S. lycopersicum, Aquilegia vulgaris, A. thaliana, Capsicum* spp, *Petunia hybrid, Solanum* spp, *Gossypium* spp, *Thalictrum dioicum, Jatropha curcas L, Nicotiana attenuate, Centaurea cyanus*	Tobravirus	Agro-inoculation, Agro-infiltration	*pds, rbcS, FLO/LFY (NFL)*	Positive-strand RNA, bipartite	[36]; [37]; [38]; [39]; [40]
**Turnip yellow mosaic virus (TYMV)**	*Arabidopsis thaliana, Brassica rapa, brassica napus,* Radish (*Raphanus sativus* L.)	Tymovirus	Rub inoculation, Gene Gun	*lfy, pds, RsNHX1*	Positive-strand RNA, monopartite	[41]; [42]; [43]
**Foxtail mosaic virus**	*Hordeum vulgare, Triticum aestivum* and *Setaria italica, Zea mays*	Potexvirus	Agro-infiltration	Several different reporter gene and *pds, CLA1, IspH, midrib3, iojap, lesion mimic22*	+ssRNA, Monopartite	[44]; [45]
**Chinese wheat mosaic virus**	*N. benthamiana* and Triticum *aestivum*	Furovirus	Agroinfiltration	*PDS,* miRNAs (miR165/166 and miR3134a)	+ssRNA	[46]
			**DNA Viruses**			
**Abutilon mosaic virus (AbMV)**	*N. benthamiana*	Begomovirus	Agro inoculation	*cpHSC70*	Single-stranded (S S)	[47]

Table S1

					DNA, Bipartite	
**African cassava mosaic virus (ACMV)**	*Manihot esculenta* and *N. benthamiana*	Begomovirus	Particle bombardment	*su, cyp79d2*	ssDNA bipartite	[48]
**Cassava common mosaic virus (CsCMV)**	cassava varieties and *Nicotiana benthamiana*	Potexvirus	Agro inoculation	*PDS* and *ChlI*	Single-stranded RNA, Monopartite	[49]
**Beet curly top virus (BCTV)**	*Spinacea oleracea* and *S. lycopersicum*	Geminivirus	Biolistic techniques, Agro infiltration	*rbcS* and *ChlI,*	Circular, single-stranded (*ss*)*DNA*	[50]
**Cabbage leaf curl virus (CaLCuV)**	*A. thaliana,* Brassica crop species (*Brassica rapa* and *Brassica nigra)* and *N. benthamiana*	Begomovirus	Particle bombardment, Agro inoculation, *Agrobacterium* syringe infiltration	*gfp, CH42, pds*	ssDNA, bipartite	[51]; [52]; [53]
**Cotton leaf crumple virus (CLCrV)**	*Gossypium* spp	Begomovirus	Particle bombardment, Agro-inoculation, and *Agrobacterium*-mediated infiltration	*ChlI*, *PDS*, *CHLI* and EF-*1*α	(*ss*)DNA, bipartite	[54]; [55]
**Grapevine virus A (GVA)**	*N. benthamiana* and *Vitis vinifera*	Vitivirus	Agro inoculation	*PDS*	Single-stranded RNA	[56]
**Pepper Huasteco yellow vein**	*Capsicum* spp	Begomovirus	Particle bombardment	*Comt, pAmt* and *Kas*	Circular, *ss*DNA bipartite	[57]

**virus (PHYV)**						
**Rice tungro bacilliform virus (RTBV)**	*O. sativa, Cynodon dactylon* and *Zoysia japonica*	Tungrovirus	Agro-inoculation, Agro-infiltration	*Pds*	Circular double-stranded DNA	[58]; [59]
**Tomato golden mosaic virus (TGMV)**	*N. benthamiana*	Begomovirus	Particle bombardment	*su, luc*	ssDNA, bipartite	[60]
**Tomato leaf curl virus (ToLCV)**	*N.benthamiana* and *S. lycopersicum*	Begomovirus	Agro inoculation	*PCNA*	Circular, ssDNA	[61]
			**RNA satellite virus**			
**Satellite tobacco mosaic virus (STMV)**	*N. tabacum*	RNA satellite Virus	Rub inoculation	*cesA-1a, cesA-2, als, chsA, rbcS, rbcL, tk*	Single stranded positive-sense RNA	[62]
**Bamboo mosaic virus (BMV)**	*B. distachyon, N. benthamiana*	Potexvirus	Rub inoculation and Agroinfiltration	*Sul* and *PDS*	RNA satellite Virus	[63]
			**DNA satellite virus**			
**Tomato yellow leaf curl china virus (TYLCCNV) Beta-satellite**	*S.lycopersicu, Nicotina spp, and Petunia hybrida*	DNA beta satellite	Agro inoculation	*Pcna, pds, su, FRO1*	ssDNA, satellite	[64]; [65]
**Bhendi yellow vein mosaic virus Beta-satellite**	*N. benthamiana*	DNA beta satellite	Inoculation with needle-less syringe	*Su, PDS, PCNA* and *AGO1*	ssDNA, satellite	[66]

Table S1

**Tobacco curly shoot virus (TbCSV) Beta-satellite**	*N.benthamiana*	Begomovirus	Agro inoculation	*gfp, su, chs, pcna*	DNA, satellite	[67]; [68]
**TbCSV Alpha-satellite**	*S.lycopersicu-m* and *Nicotina spp, Petunia hybrid*	Begomovirus	Agro inoculation	*β-glucuronidase* (*GUS*) gene, *Su*, *NtEDS1*	*Circular, ssDNA* monopartite	[68]; [69]

References

[1] M.H. Kumagai, J. Donson, G. Della-Cioppa, D. Harvey, K. Hanley, L.J.P.o.t.N.A.o.S. Grill, Cytoplasmic inhibition of carotenoid biosynthesis with virus-derived RNA, 92(5) (1995) 1679-1683.

[2] V. Gosselé, I. Faché, F. Meulewaeter, M. Cornelissen, M.J.T.P.J. Metzlaff, SVISS–a novel transient gene silencing system for gene function discovery and validation in tobacco plants, 32(5) (2002) 859-866.

[3] J.-B. Hiriart, E.-M. Aro, K.J.M.p.-m.i. Lehto, Dynamics of the VIGS-mediated chimeric silencing of the Nicotiana benthamiana ChlH gene and of the tobacco mosaic virus vector, 16(2) (2003) 99-106.

[4] A. Igarashi, K. Yamagata, T. Sugai, Y. Takahashi, E. Sugawara, A. Tamura, H. Yaegashi, N. Yamagishi, T. Takahashi, M.J.V. Isogai, Apple latent spherical virus vectors for reliable and effective virus-induced gene silencing among a broad range of plants including tobacco, tomato, Arabidopsis thaliana, cucurbits, and legumes, 386(2) (2009) 407-416.

[5] N. Yamagishi, N.J.P.m.b. Yoshikawa, Virus-induced gene silencing in soybean seeds and the emergence stage of soybean plants with Apple latent spherical virus vectors, 71(1) (2009) 15-24.

[6] N. Yamagishi, N.J.J.o.V.M. Yoshikawa, Efficient virus-induced gene silencing system in pumpkin (Cucurbita maxima) using apple latent spherical virus vector, 301 (2022) 114456.

[7] X.S. Ding, W.L. Schneider, S.R. Chaluvadi, M.R. Mian, R.S.J.M.P.-M.I. Nelson, Characterization of a Brome mosaic virus strain and its use as a vector for gene silencing in monocotyledonous hosts, 19(11) (2006) 1229-1239.

[8] S.R. Scofield, R.S.J.P.p. Nelson, Resources for virus-induced gene silencing in the grasses, 149(1) (2009) 152-157.

[9] K. van der Linde, C. Kastner, J. Kumlehn, R. Kahmann, G.J.N.P. Doehlemann, Systemic virus-induced gene silencing allows functional characterization of maize genes during biotrophic interaction with Ustilago maydis, 189(2) (2011) 471-483.

[10] X.S. Ding, S.W. Mannas, B.A. Bishop, X. Rao, M. Lecoultre, S. Kwon, R.S.J.P.P. Nelson, An improved brome mosaic virus silencing vector: greater insert stability and more extensive VIGS, 176(1) (2018) 496-510.

[11] Y. Wang, C. Chai, B. Khatabi, W.-R. Scheible, M.K. Udvardi, M.C. Saha, Y. Kang, R.S.J.F.i.p.s. Nelson, An Efficient Brome mosaic virus-based gene silencing protocol for hexaploid wheat (Triticum aestivum L.), 12 (2021) 1036.

[12] S. Holzberg, P. Brosio, C. Gross, G.P.J.T.P.J. Pogue, Barley stripe mosaic virus-induced gene silencing in a monocot plant, 30(3) (2002) 315-327. 13] M. Bruun-Rasmussen, C.T. Madsen, S. Jessing, M.J.M.P.-M.I. Albrechtsen, Stability of barley stripe mosaic virus–induced gene silencing in barley, 20(11) (2007) 1323-1331.

[14] A. Pacak, K. Geisler, B. Jørgensen, M. Barciszewska-Pacak, L. Nilsson, T.H. Nielsen, E. Johansen, M. Grønlund, I. Jakobsen, M.J.P.m. Albrechtsen, Investigations of barley stripe mosaic virus as a gene silencing vector in barley roots and in Brachypodium distachyon and oat, 6(1) (2010) 1-16.

[15] C. Yuan, C. Li, L. Yan, A.O. Jackson, Z. Liu, C. Han, J. Yu, D. Li, A high throughput barley stripe mosaic virus vector for virus induced gene silencing in monocots and dicots, PLoS One 6(10) (2011) e26468.

[16] C.S. Gordon, N. Rajagopalan, E.P. Risseeuw, M. Surpin, F.J. Ball, C.J. Barber, L.M. Buhrow, S.M. Clark, J.E. Page, C.D.J.P.O. Todd, Characterization of Triticum aestivum abscisic acid receptors and a possible role for these in mediating Fusairum head blight susceptibility in wheat, 11(10) (2016) e0164996.

[17] C. Zhang, S.A.J.V. Ghabrial, Development of Bean pod mottle virus-based vectors for stable protein expression and sequence-specific virus-induced gene silencing in soybean, 344(2) (2006) 401-411.

[18] C. Zhang, C. Yang, S.A. Whitham, J.H.J.M.p.-m.i. Hill, Development and use of an efficient DNA-based viral gene silencing vector for soybean, 22(2) (2009) 123-131.

[19] C. Zhang, J.D. Bradshaw, S.A. Whitham, J.H.J.P.p. Hill, The development of an efficient multipurpose bean pod mottle virus viral vector set for foreign gene expression and RNA silencing, 153(1) (2010) 52-65.

[20] Q. Ren, H. Jiang, W. Xiang, Y. Nie, S. Xue, H. Zhi, K. Li, J.J.T.C.J. Gai, A MADS-box gene is involved in soybean resistance to multiple soybean mosaic virus strains, 10(3) (2022) 802-808.

[21] A. Nagamatsu, C. Masuta, M. Senda, H. Matsuura, A. Kasai, J.S. Hong, K. Kitamura, J. Abe, A.J.P.b.j. Kanazawa, Functional analysis of soybean genes involved in flavonoid biosynthesis by virus-induced gene silencing, 5(6) (2007) 778-790.

[22] H. Li, D. Zhang, K. Xie, Y. Wang, Q. Liao, Y. Hong, Y.J.P.P. Liu, Efficient and high-throughput pseudorecombinant-chimeric Cucumber mosaic virus-based VIGS in maize, 187(4) (2021) 2865-2876.

[23] H.-C. Lu, H.-H. Chen, W.-C. Tsai, W.-H. Chen, H.-J. Su, D.C.-N. Chang, H.-H.J.P.P. Yeh, Strategies for functional validation of genes involved in reproductive stages of orchids, 143(2) (2007) 558-569.

[24] M.-H. Hsieh, H.-C. Lu, Z.-J. Pan, H.-H. Yeh, S.-S. Wang, W.-H. Chen, H.-H.J.P.S. Chen, Optimizing virus-induced gene silencing efficiency with Cymbidium mosaic virus in Phalaenopsis flower, 201 (2013) 25-41.

[25] M.T. Ruiz, O. Voinnet, D.C.J.T.P.C. Baulcombe, Initiation and maintenance of virus-induced gene silencing, 10(6) (1998) 937-946.

[26] C. Burger, S. Rondet, P. Benveniste, H.J.J.o.e.b. Schaller, Virus-induced silencing of sterol biosynthetic genes: identification of a Nicotiana tabacum L. obtusifoliol-14α-demethylase (CYP51) by genetic manipulation of the sterol biosynthetic pathway in Nicotiana benthamiana L, 54(388) (2003) 1675-1683.

[27] O. Faivre-Rampant, E.M. Gilroy, K. Hrubikova, I. Hein, S. Millam, G.J. Loake, P. Birch, M. Taylor, C.J.P.P. Lacomme, Potato virus X-induced gene silencing in leaves and tubers of potato, 134(4) (2004) 1308-1316.

[28] E. Gammelgård, M. Mohan, J.P.J.J.o.g.v. Valkonen, Potyvirus-induced gene silencing: the dynamic process of systemic silencing and silencing suppression, 88(8) (2007) 2337-2346.

[29] F. Houhou, Biotechnological application of potyvirus-based vectors in cucurbit plants, Universitat Politècnica de València, 2021.

[30] M. Grønlund, G. Constantin, E. Piednoir, J. Kovacev, I.E. Johansen, O.S.J.V.R. Lund, Virus-induced gene silencing in Medicago truncatula and Lathyrus odorata, 135(2) (2008) 345-349.

[31] M. Naylor, J. Reeves, J. Cooper, M.-L. Edwards, H.J.J.o.v.m. Wang, Construction and properties of a gene-silencing vector based on Poplar mosaic virus (genus Carlavirus), 124(1-2) (2005) 27-36.

[32] F.E. Vaistij, L.J.P.p. Jones, Compromised virus-induced gene silencing in RDR6-deficient plants, 149(3) (2009) 1399-1407.

[33] É. Várallyay, Z. Lichner, J. Sáfrány, Z. Havelda, P. Salamon, G. Bisztray, J.J.A.B.H. Burgyán, Development of a virus induced gene silencing vector from a legumes infecting tobamovirus, 61(4) (2010) 457-469.

[34] Y. Gao, Y.-L. Zhang, X.-F. Zhang, C.-G. Han, J.-L. Yu, D.-W. Li, Development and optimization of Tobacco necrosis virus A induced gene silencing in Nicotiana benthamiana, Progress in Biochemistry and Biophysics 38(10) (2011) 919-928.

[35] D. Pignatta, P. Kumar, M. Turina, A. Dandekar, B.W. Falk, Quantitative analysis of efficient endogenous gene silencing in Nicotiana benthamiana plants using tomato bushy stunt virus vectors that retain the capsid protein gene, Molecular plant-microbe interactions 20(6) (2007) 609-618.

[36] F. Ratcliff, A.M. Martin-Hernandez, D.C. Baulcombe, Technical advance: tobacco rattle virus as a vector for analysis of gene function by silencing, The Plant Journal 25(2) (2001) 237-245.

[37] Y. Liu, M. Schiff, R. Marathe, S. Dinesh-Kumar, Tobacco Rar1, EDS1 and NPR1/NIM1 like genes are required for N-mediated resistance to tobacco mosaic virus, The Plant Journal 30(4) (2002) 415-429.

[38] V.S. Di Stilio, Empowering plant evo-devo: Virus induced gene silencing validates new and emerging model systems, BioEssays 33(9) (2011) 711-718.

[39] C. Deng, F. Zhang, J. Wang, Y. Li, H. Huang, S. Dai, Tobacco Rattle Virus-induced Phytoene Desaturase (PDS) Silencing in Centaurea cyanus, Horticultural Plant Journal 7(2) (2021) 159-166.

[40] J. Rahman, I.T. Baldwin, K. Gase, California TRV-based VIGS vectors mediate gene silencing at elevated temperatures but with greater growth stunting, BMC plant biology 21(1) (2021) 1-17.

[41] S. Pflieger, S. Blanchet, L. Camborde, G. Drugeon, A. Rousseau, M. Noizet, S. Planchais, I. Jupin, Efficient virus-induced gene silencing in Arabidopsis using a 'one-step'TYMV-derived vector, The Plant Journal 56(4) (2008) 678-690.

[42] J. Yu, X.-D. Yang, Q. Wang, L.-W. Gao, Y. Yang, D. Xiao, T.-K. Liu, Y. Li, X.-L. Hou, C.-W. Zhang, Efficient virus-induced gene silencing in Brassica rapa using a turnip yellow mosaic virus vector, Biologia plantarum 62(4) (2018) 826-834.

[43] Y. Wang, J. Ying, Y. Zhang, L. Xu, W. Zhang, M. Ni, Y. Zhu, L. Liu, Genome-wide identification and functional characterization of the cation proton antiporter (CPA) family related to salt stress response in radish (Raphanus sativus L.), International journal of molecular sciences 21(21) (2020) 8262.

[44] N. Liu, K. Xie, Q. Jia, J. Zhao, T. Chen, H. Li, X. Wei, X. Diao, Y. Hong, Y. Liu, Foxtail mosaic virus-induced gene silencing in monocot plants, Plant physiology 171(3) (2016) 1801-1807.

[45] Y. Mei, C. Zhang, B.M. Kernodle, J.H. Hill, S.A. Whitham, A Foxtail mosaic virus vector for virus-induced gene silencing in maize, Plant Physiology 171(2) (2016) 760-772.

[46] J. Yang, T.-Y. Zhang, Q.-S. Liao, L. He, J. Li, H.-M. Zhang, X. Chen, J. Li, J. Yang, J.-B. Li, Chinese wheat mosaic virus-induced gene silencing in monocots and dicots at low temperature, Frontiers in plant science 9 (2018) 1627.

[47] B. Krenz, C. Wege, H. Jeske, Cell-free construction of disarmed Abutilon mosaic virus-based gene silencing vectors, Journal of virological methods 169(1) (2010) 129-137.

[48] I.B. Fofana, A. Sangaré, R. Collier, C. Taylor, C.M. Fauquet, A geminivirus-induced gene silencing system for gene function validation in cassava, Plant molecular biology 56(4) (2004) 613-624.

[49] D. Tuo, P. Zhou, P. Yan, H. Cui, Y. Liu, H. Wang, X. Yang, W. Liao, D. Sun, X. Li, A cassava common mosaic virus vector for virus-induced gene silencing in cassava, Plant Methods 17(1) (2021) 1-12.

[50] E.M. Golenberg, D. Sather, L.C. Hancock, K.J. Buckley, N.M. Villafranco, D.M. Bisaro, Development of a gene silencing DNA vector derived from a broad host range geminivirus, Plant Methods 5(1) (2009) 1-14.

[51] M.A. Turnage, N. Muangsan, C.G. Peele, D. Robertson, Geminivirus-based vectors for gene silencing in Arabidopsis, The Plant Journal 30(1) (2002) 107-114.

[52] C. Huang, Y. Qian, Z. Li, X. Zhou, Virus-induced gene silencing and its application in plant functional genomics, Science China Life Sciences 55(2) (2012) 99-108.

[53] Z. Xiao, M. Xing, X. Liu, Z. Fang, L. Yang, Y. Zhang, Y. Wang, M. Zhuang, H. Lv, An efficient virus-induced gene silencing (VIGS) system for functional genomics in Brassicas using a cabbage leaf curl virus (CaLCuV)-based vector, Planta 252(3) (2020) 1-12.

[54] J.R. Tuttle, A. Idris, J.K. Brown, C.H. Haigler, D. Robertson, Geminivirus-mediated gene silencing from Cotton leaf crumple virus is enhanced by low temperature in cotton, Plant Physiology 148(1) (2008) 41-50.

[55] Z. Gu, C. Huang, F. Li, X. Zhou, A versatile system for functional analysis of genes and micro RNA s in cotton, Plant biotechnology journal 12(5) (2014) 638-649.

[56] M. Muruganantham, Y. Moskovitz, S. Haviv, T. Horesh, A. Fenigstein, J. du Preez, D. Stephan, J.T. Burger, M. Mawassi, Grapevine virus A-mediated gene silencing in Nicotiana benthamiana and Vitis vinifera, Journal of virological methods 155(2) (2009) 167-174.

[57] M. del Rosario Abraham-Juárez, M. del Carmen Rocha-Granados, M.G. López, R.F. Rivera-Bustamante, N. Ochoa-Alejo, Virus-induced silencing of Comt, pAmt and Kas genes results in a reduction of capsaicinoid accumulation in chili pepper fruits, Planta 227(3) (2008) 681-695.

[58] A. Purkayastha, S. Mathur, V. Verma, S. Sharma, I. Dasgupta, Virus-induced gene silencing in rice using a vector derived from a DNA virus, Planta 232(6) (2010) 1531-1540.

[59] B. Zhang, J.-A. Shi, J.-B. Chen, D.-D. Li, J.-J. Li, H.-L. Guo, J.-Q. Zong, Y. Wang, A.-G. Guo, J.-X. Liu, Efficient virus-induced gene silencing in Cynodon dactylon and Zoysia japonica using rice tungro bacilliform virus vectors, Scientia Horticulturae 207 (2016) 97-103.

[60] J. Carrillo-Tripp, H. Shimada-Beltrán, R. Rivera-Bustamante, Use of geminiviral vectors for functional genomics, Current opinion in plant biology 9(2) (2006) 209-215.

[61] P. Pandey, N.R. Choudhury, S.K. Mukherjee, A geminiviral amplicon (VA) derived from Tomato leaf curl virus (ToLCV) can replicate in a wide variety of plant species and also acts as a VIGS vector, Virology Journal 6(1) (2009) 1-13.

[62] V. Gosselé, I. Faché, F. Meulewaeter, M. Cornelissen, M. Metzlaff, SVISS–a novel transient gene silencing system for gene function discovery and validation in tobacco plants, The Plant Journal 32(5) (2002) 859-866.

[63] M.R. Liou, Y.W. Huang, C.C. Hu, N.S. Lin, Y.H. Hsu, A dual gene-silencing vector system for monocot and dicot plants, Plant Biotechnology Journal 12(3) (2014) 330-343.

[64] X. Tao, X. Zhou, A modified viral satellite DNA that suppresses gene expression in plants, The Plant Journal 38(5) (2004) 850-860.

[65] X. He, C. Jin, G. Li, G. You, X. Zhou, S. Zheng, Use of the modified viral satellite DNA vector to silence mineral nutrition-related genes in plants: silencing of the tomato ferric chelate reductase gene, FRO1, as an example, Science in China Series C: Life Sciences 51(5) (2008) 402-409.

[66] C. Jeyabharathy, H. Shakila, R. Usha, Development of a VIGS vector based on the β-satellite DNA associated with bhendi yellow vein mosaic virus, Virus research 195 (2015) 73-78.

[67] Y. Qian, R.B. Mugiira, X. Zhou, A modified viral satellite DNA-based gene silencing vector is effective in association with heterologous begomoviruses, Virus research 118(1-2) (2006) 136-142.

[68] C. Huang, Y. Xie, X. Zhou, Efficient virus-induced gene silencing in plants using a modified geminivirus DNA1 component, Plant biotechnology journal 7(3) (2009) 254-265.

[69] C.-j. Huang, T. Zhang, F.-f. Li, X.-y. Zhang, X.-p. Zhou, Development and application of an efficient virus-induced gene silencing system in Nicotiana tabacum using geminivirus alphasatellite, Journal of Zhejiang University Science B 12(2) (2011) 83-92.
